# The circRNA circIFI30 promotes progression of triple-negative breast cancer and correlates with prognosis

**DOI:** 10.18632/aging.103311

**Published:** 2020-06-04

**Authors:** Lei Xing, Rui Yang, Xiaosong Wang, Xiaying Zheng, Xin Yang, Luyu Zhang, Rong Jiang, Guosheng Ren, Junxia Chen

**Affiliations:** 1 Department of Endocrine and Breast Surgery, The First Affiliated Hospital, Chongqing Medical University, Chongqing, China; 2 Department of Cell Biology and Genetics, Chongqing Medical University, Chongqing, China; 3 Department of Thoracic Surgery, The First Affiliated Hospital, Chongqing Medical University, Chongqing, China; 4 Molecular Medicine and Cancer Research Center, Chongqing Medical University, Chongqing, China; 5 Laboratory of Stem Cells and Tissue Engineering, Chongqing Medical University, Chongqing, China

**Keywords:** circIFI30, miR-520b-3p, triple-negative breast cancer, CD44

## Abstract

Growing evidence suggests that circRNAs exert a critical role in tumorigenesis and cancer progression. To date, the molecular mechanisms underlying circRNAs in triple-negative breast cancer (TNBC) are still poorly known. Here, circRNA expression profile was investigated by RNA sequencing in TNBC tissues and matched para-carcinoma tissues. We found that circIFI30 was significantly up-regulated in TNBC tissues and cells using quantitative real-time PCR and in situ hybridization. High circIFI30 expression was positively correlated with clinical TNM stage, pathological grade and poor prognosis of TNBC patients. Functionally, a series of *in vivo* and *in vitro* experiments showed that knockdown of circIFI30 could markedly inhibit TNBC cell proliferation, migration, invasion and cell cycle progression, induce apoptosis as well as suppress tumorigenesis and metastasis. Up-regulation of circIFI30 exerted an opposite effect. Mechanistically, we demonstrated that circIFI30 might act as a competing endogenous RNA (ceRNA) of miR-520b-3p to abolish the suppressive effect on target gene CD44 by fluorescent in situ hybridization (FISH), dual luciferase reporter assay, RNA immunoprecipitation and RNA pull-down assays. Therefore, our work uncovers the mechanism by which circIFI30 could promote TNBC progression through circIFI30/miR-520b-3p/CD44 axis and circIFI30 could be a novel diagnostic/prognostic marker and therapeutic target for TNBC patients.

## INTRODUCTION

Breast cancer is the most common female cancer, new cases and deaths for breast cancer were 2,088,849 and 626,679, accounting for almost 1 in 4 cancer cases among women in 2018 around the world according to Global Cancer Statistics [1]. Despite the early detection and efficient systemic treatment, breast cancer is also the leading cause of cancer death in over 100 countries. Breast cancer is a heterogeneous disease including four main subtypes. Triple-negative breast cancer (TNBC) is a breast cancer subtype with negative expressions of estrogen receptor (ER), progesterone receptors (PR) and human epidermal growth factor receptor 2 (HER2). TNBC has special biological and clinicopathological characteristics such as strong proliferation and invasion capabilities, high recurrence and metastasis rate and poor prognosis. The median survival time of patients with metastatic TNBC was only 13.3 months [2]. TNBC patients lack effective endocrine therapy and anti-HER2 targeted therapy because of no expression of ER, PR and HER2. So far, chemotherapy remains the main treatment for TNBC [3]. Therefore, investigating the molecular mechanisms underlying tumorigenesis and development of TNBC as well as finding the effective potential target are of great significance for improving the survival and prognosis for TNBC patients.

Circular RNAs (circRNAs), a kind of noncoding RNA molecules, are characterized by a covalently closed continuous loop without 5′-3′ polarity and poly A tail. Because of this unique structure, circRNAs are stable in the cells and are not easily degraded by exonuclease RNase R. Currently, circRNAs were known to be abundant, conserved and tissue/developmental-stage specific. Recent studies have shown that circRNAs play an important potential role in the regulation of gene expression [4]. Accumulating evidence indicates that circRNAs play important roles in the pathogenesis and progression of many cancers. Some circRNAs such as circHIPK3, circFBLIM1 and circABCB10 have shown great potential in diagnosis, therapy and prognosis for bladder cancer, hepatocellular cancer and breast cancer [5–7]. However, the roles of circRNAs in the development of TNBC have been rarely reported to date, the biological functions and underlying mechanisms of most circRNAs have not been explored.

MicroRNAs (miRNAs), a class of evolutionary conserved small non-coding RNAs, are regulator of gene expression at the post transcription level via targeting mRNAs, leading to mRNA degradation or translation inhibition [8]. The upregulation of oncogenic miRNAs results in a decrease in the expression of tumor suppressor genes. Conversely, the downregulation of tumor-suppressive miRNAs increases the expression of oncogenes [9]. Recently, Pandolfi et al. proposed the competitive endogenous RNA (ceRNA) hypothesis that lncRNAs, mRNAs and pseudogenes could regulate each other's expression through competing for shared miRNAs, providing a new mechanism for regulation of gene expression [10]. Researches showed that more than 80% of circRNAs are derived from exons and have identical sequences with the corresponding linear mRNA. Thus, circRNA might function as a new member of ceRNA family and modulators of miRNA activity by competing for common miRNA binding sites, which plays an important role in regulating gene expression in tumors and other diseases [11]. Increasing evidence showed that circRNAs could serve as ceRNAs to be implicated in the progression of breast cancer, gastric cancer, colorectal cancer, bladder cancer and hepatocellular carcinoma [12–16]. However, underlying mechanisms of circRNAs in tumorigenesis and cancer progression including TNBC remain largely unknown.

Here, the expression profile of circRNAs was detected in TNBC utilizing RNA-seq and characterized a new circRNA termed circIFI30 from IFI30 gene with a circBase ID of hsa_circ_0005571. We then investigated the clinicopathological significance of circIFI30 expression and explored the function as well as the underlying molecular mechanism of circIFI30 in TNBC progression. We found that circIFI30 was significantly upregulated in TNBC and correlated associated with pathological grade, clinical stage and poor prognosis. Further research demonstrated that circIFI30 could function as a sponge for miR-520b-3p to promote cell proliferation, invasion, tumor growth and metastasis through up-regulating the expression of miR-520b-3p target gene CD44. Therefore, circIFI30 might be a novel prognostic biomarker and therapeutic target for TNBC.

## RESULTS

### circRNA expression profile and circIFI30 are identified in TNBC

**Figure 1:**
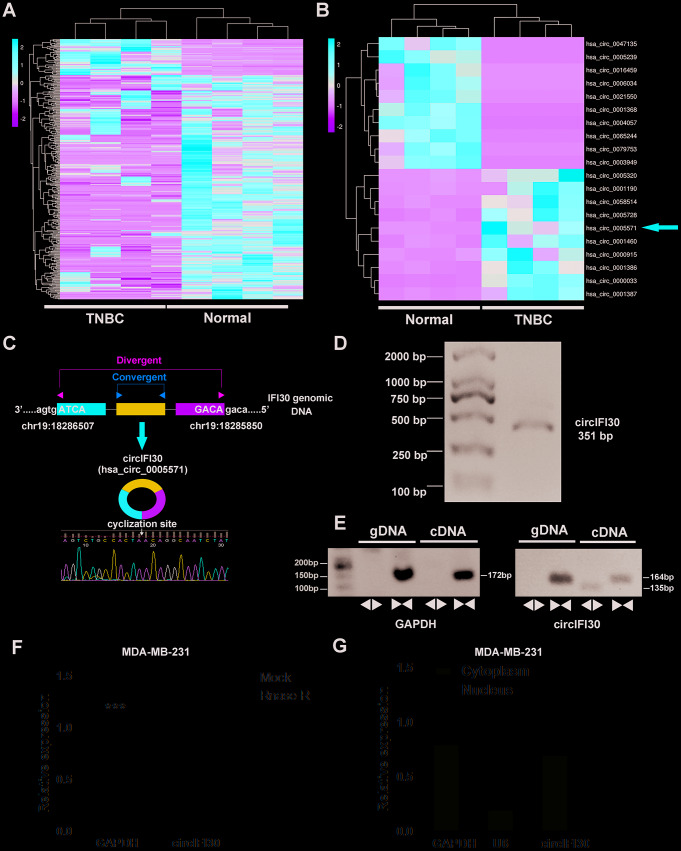
**Expression profile of circRNA in TNBC and para-carcinoma tissues by RNA sequencing and characterization of circIFI30.** (**A**) Hierarchical cluster analysis of all target circRNAs in the TNBC and matched para-carcinoma tissues was shown. Each column represents a sample and each row represents a circRNA. Red strip represents high relative expression and green strip represents low relative expression. (**B**) The cluster heat map showed the top 10 up-regulated and down-regulated circRNAs. (**C**) The genomic locus of the circIFI30 and the back-spliced junction of circIFI30 were indicated, the back-splice junction sequence was validated by Sanger sequencing. (**D**) PCR product of circIFI30 was confirmed by agarose gel electrophoresis. (**E**) CircIFI30, linear IFI30 and GAPDH were amplified from cDNA or gDNA in MDA-MB-231 cells with divergent and convergent primers, respectively. Divergent primers amplified circIFI30 in cDNA but not genomic DNA (gDNA). (**F**) RNase R treatment was used to evaluate the exonuclease resistance of circIFI30 in MDA-MB-231 cells. GAPDH was measured as a control. (**G**) Nuclear-cytoplasmic fractionation assay showed that circIFI30 was mainly localized in the cytoplasm of MDA-MB-231 cells. GAPDH was considered as a cytoplasmic control. U6 was used as a nuclear control.

To identify and characterize differentially expressed circRNAs in TNBC, RNA-seq was implemented in 4 pairs of TNBC and adjacent noncancerous tissues. The result showed that 354 circRNAs were significantly differentially expressed in TNBC tissues compared with paired adjacent normal tissues, of which 47 were upregulated and 307 were downregulated (Figure 1A). The top 20 dysregulated circRNAs were indicated with heatmap (Figure 1B). Interestingly, we discovered that circIFI30 (hsa_circ_0005571) was one of the most significantly up-regulated circRNAs in TNBC. The circIFI30 with a length of 351 nt is produced by back-splicing of the exon 1-3 of IFI30 gene on chr19: 18286507-18285850 according to the annotation of circBase (http://www.circbase.org/), and its junction sequence was validated by sanger sequencing (Figure 1C). PCR product of circIFI30 in 293T cells was verified by agarose gel electrophoresis (Figure 1D). To confirm that circIFI30 is generated from the head-to-tail splicing instead of trans-splicing or genomic rearrangements, the divergent and convergent primers were designed to amplify circIFI30 circular transcripts and IFI30 linear transcripts, respectively. PCR results showed that circIFI30 was only detected in cDNA, whereas the convergent primers amplified linear IFI30 from both cDNA and gDNA (Figure 1E). Moreover, the experiment showed that a 3’ to 5’ exoribonuclease named RNase R could rapidly degrade linear GAPDH rather than circIFI30 (Figure 1F). Furthermore, we detected the subcellular localization of circIFI30 by nuclear-cytoplasmic fractionation assay. The results showed that circIFI30 was mainly distributed in the cytoplasm of TNBC cells (Figure 1G). These data suggested that circIFI30 is a circular RNA and could function as a miRNA sponge.

### circIFI30 is highly expressed in TNBC and correlated with pathological stage and poor prognosis

**Figure 2:**
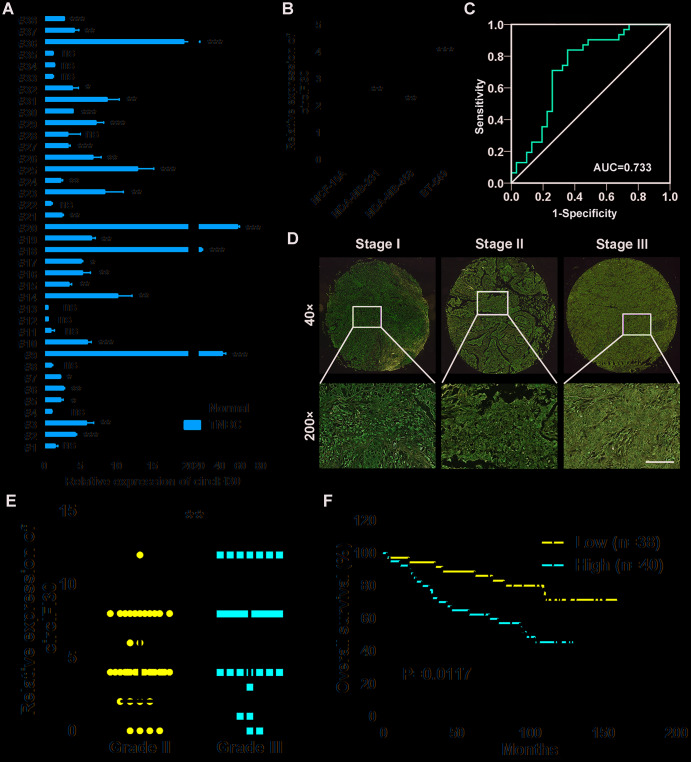
**circIFI30 is up-regulated in TNBC and associated with the progression and poor prognosis of TNBC patients.** (**A**) Relative expression of circIFI30 in TNBC tissues and adjacent non-tumor tissues was detected by qRT-PCR (n = 38). (**B**) Relative expression of circIFI30 in cell lines was determined by qRT-PCR. (**C**) ROC curve was applied to evaluate the diagnostic value of circIFI30 for TNBC. (**D**) Representative images of circIFI30 expression in TNBC tissues were detected by ISH assays. Scale bar, 100 μm. (**E**) Dot distribution graph of circIFI30 ISH staining scores was shown in TNBC patients with different pathological grades. (**F**) Kaplan-Meier survival curve of overall survival in 78 patients with TNBC according to the circIFI30 expression. Patients were stratified into high expression and low expression group by median expression. Data were showed as mean ± SD, *P < 0.05, **P < 0.01, ***P < 0.001.

To determine the expression and clinical value of circIFI30, qRT-PCR was executed to detect the expression levels of circIFI30 in 38 pairs of TNBC tissues and adjacent non-cancerous tissues, TNBC cell lines (MDA-MB-231, MDA-MB-468 and BT-549) and normal breast epithelial cells (MCF-10A). Consistent with our RNA-seq results, circIFI30 was significantly up-regulated in TNBC tissues and cell lines (Figure 2A, 2B). Receiver Operating Characteristic (ROC) analysis displayed that circIFI30 could sensitively distinguish TNBC tissues from noncancerous tissues (Figure 2C). circIFI30 expression was then detected by ISH on tissue microarrays **(**TMAs) with 78 TNBC tissues (Figure 2D, 2E). The correlation between circIFI30 expression and clinical characteristics in patients with TNBC (cohort2) was showed in Table 1. The expression of circIFI30 was significantly correlated with age (P=0.028), histological grade (P=0.003) and clinical stage (P=0.012). Kaplan-Meier survival analysis showed that the expression of circIFI30 was negatively related to overall survival of TNBC patients (Figure 2F). The expression level of circIFI30 was an independent prognostic factor in TNBC patients by multivariate Cox regression analysis (Table 2). These data confirmed our RNA-seq results and suggested that circIFI30 could be involved in the pathogenesis and progression of TNBC.

**Table 1:** Correlation between circIFI30 expression and clinicopathological features in 78 TNBC patients (cohort 2).

**Characteristic**		**Total**	**circIFI in cancer**		**Chi-square**	***P***
			**Low**	**High**		
**Age**	≥50	50	29	21	4.803	0.028*
	<50	28	9	19		
**Histological grade**	II	40	26	14	8.712	0.003*
	III	38	12	26		
**T stage**	T1-2	68	33	35	0.053	0.818
	T3-4	9	4	5		
**N stage**	N0	43	23	20	1.153	0.283
	N1-3	34	14	20		
**Clinical stage**	I- II	54	31	23	6.339	0.012*
	III-IV	23	6	17		

*, *P*<0.05

**Table 2:** Multivariate Cox regression analysis of circIFI30 and survival in patients with TNBC (cohort 2).

**Clinical variables**	**HR**	**95%CI**	***P***
Age (≥50 vs. <50)	1.613	0.686-3.792	0.273
Grade (II vs. III)	0.539	0.229-1.265	0.155
T stage (T1 vs. T2/3)	1.887	0.633-5.627	0.255
N stage (N0 vs. N1-3)	1.789	0.553-5.783	0.331
TNM stage (I-II vs. III)	1.064	0.300-3.771	0.924
CircIFI30 (low vs. high)	3.621	1.371-9.563	0.009*

Abbreviations: *HR* hazard ratio, *CI* confidence interval. *, *P*<0.05.

### circIFI30 enhances proliferation of TNBC cells

**Figure 3:**
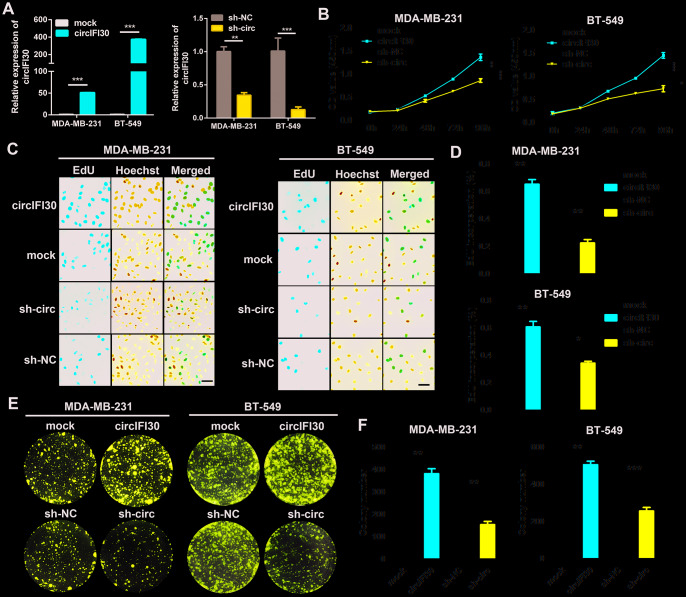
**circIFI30 promotes TNBC cell proliferation.** (**A**) Relative expression of circIFI30 was determined in TNBC cells transfected with circIFI30 expression vector, mock, sh-circ or sh-NC by qRT-PCR. (**B**) The cell viability was measured in TNBC cells transfected with indicated vectors by CCK-8 assay. (**C**, **D**) The cell proliferation ability was detected in TNBC cells after transfection with indicated plasmids by EdU assay. Scale bar, 50 μm. (**E**, **F**) Cell survival was evaluated in TNBC cells transfected with indicated plasmids by colony formation assay. Data were showed as mean ± SD, *P < 0.05, **P < 0.01, ***P < 0.001.

To probe the biological function of circIFI30 in TNBC cells, we constructed the overexpression and the RNAi vectors of circIFI30. The results showed that circIFI30 was significantly up-regulated or downregulated in TNBC cells transfected with overexpression or RNAi plasmids by qRT-PCR (Figure 3A). The growth curves revealed that up-regulation of circIFI30 significantly increased the proliferation activity of TNBC cells, whereas downregulation of circIFI30 suppressed the growth of TNBC cells by CCK8 assays (Figure 3B). Moreover, EdU assay displayed that overexpression of circIFI30 significantly enhanced the percentage of EdU-positive cells, whereas knockdown of circIFI30 caused the opposite effect (Figure 3C, 3D). Colony formation assay further indicated that upregulation of circIFI30 could markedly increase the viability of TNBC cells and down-regulation of circIFI30 obviously decreased growth of TNBC cells (Figure 3E, 3F). These experiments revealed that circIFI30 promoted proliferation of TNBC cells.

### circIFI30 promotes migration and invasion and regulates cell cycle and apoptosis of TNBC cells

**Figure 4:**
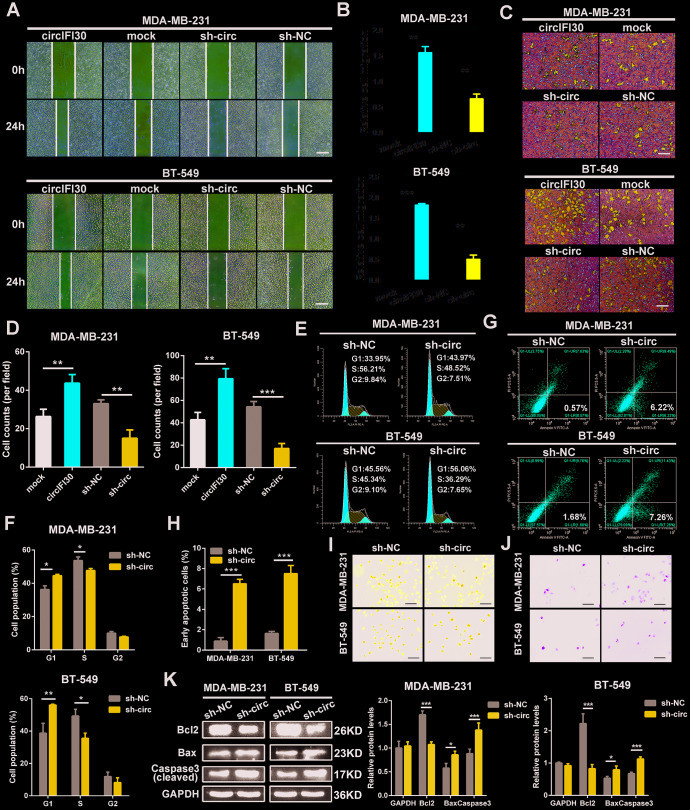
**circIFI30 increases TNBC cell migration and invasion and modulates cell cycle and apoptosis.** (**A**, **B**) The cell migration capacity was detected by wound healing assay after transfection with indicated vectors (magnification, × 50). Scale bar, 200 μm. (**C**, **D**) The cell invasion ability was determined by transwell assay after overexpression or knockdown of circIFI30 (magnification, × 100). Scale bar, 100 μm. (**E**, **F**) The cell cycle progression was analyzed by flow cytometry after transfected with indicated plasmids. (**G**, **H**) The apoptosis rate detected by flow cytometry after downregulation of circIFI30. (**I**, **J**) The apoptotic cells were observed by Hoechst 33342 (magnification, ×100, scale bar, 100 μm) and TUNEL staining (magnification, × 100, scale bar, 100 μm) assays after knockdown of circIFI30. (**K**) The expression levels of apoptosis-related proteins were determined by western blot. Data were showed as mean ± SD, *P < 0.05, **P < 0.01.

The effects of circIFI30 on migration and invasion of TNBC cells were assessed by wound healing and transwell assays. The results showed that the invasive and migratory abilities of TNBC cells were significantly increased by circIFI30 overexpression but remarkably inhibited by silencing of circIFI30 (Figure 4A–4D). Cell cycle analysis showed that downregulation of circIFI30 increased percentages of cells in G1 phase and decreased the percentages of cells in S phase compared to control group, suggesting that knockdown of circIFI30 led to cell cycle arrest at G1 in TNBC cells (Figure 4E, 4F). The apoptosis rates of cells in sh-circ group were higher than those in sh-NC control group by flow cytometry with annexin V/PI double-staining (Figure 4G, 4H). Furthermore, TNBC cells transfected with sh-circ displayed obvious morphological feature of apoptosis, such as nuclear fragment, stronger fluorescence, chromatin aggregation and apoptosis body by hoechst 33342 staining (Figure 4I). Compared with the control group, knockdown of circIFI30 remarkably enhanced the number of TUNEL-positive cells using TUNEL assay (Figure 4J). Moreover, western blot analysis indicated that the expressions of proapoptotic protein Bax and cleaved caspase-3 were increased and the level of Bcl-2 was reduced in TNBC cells after knockdown of circIFI30 compared with the control group (Figure 4K). These results further demonstrated that circIFI30 could play a vital role of in the motility and viability of TNBC cells *in vitro*.

### circIFI30 facilitates the growth and metastasis of xenograft tumors in vivo

**Figure 5:**
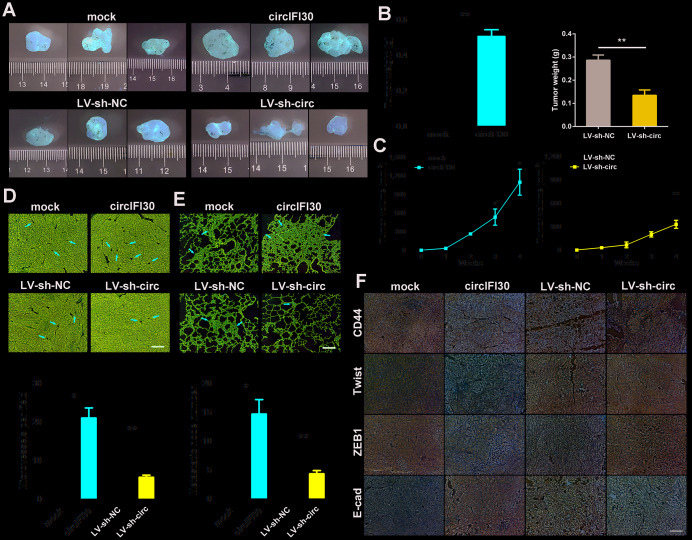
**circIFI30 facilitates tumorigenesis and metastasis of TNBC cells in vivo.** (**A**, **B**) Representative images of xenograft tumors of each group and tumor weight analysis were shown. (**C**) Growth curves of xenograft tumors were measured once a week. (**D**, **E**) HE staining of tumor and lung sections were showed. The microvessels of the tumors and metastatic nodules of the lungs were indicated by arrows (magnification, × 100, scale bar, 100 μm). (**F**) IHC staining was applied to analyze the protein levels of CD44 and EMT-related molecules (magnification, × 200, scale bar, 100 μm). Data were indicated as mean ± SD, *P < 0.05, **P < 0.01, ***P < 0.001.

To value the influence of circIFI30 on tumor growth and metastasis, female nude mice were subcutaneously inoculated with the MDA-MB-231 cells transfected stably with circIFI30 overexpression vectors or infected with lentiviruses expressing circIFI30 shRNA and their controls. The results displayed that the tumor volume and weight in overexpression circIFI30 group were obviously higher than those in the control group, while circIFI30 knockdown markedly inhibited the tumor growth (Figure 5A–5C). In addition, the overexpression of circIFI30 could remarkably promote tumor angiogenesis, whereas circIFI30 silencing significantly decreased the density of microvessels in the tumors (Figure 5D). Furthermore, the upregulation of circIFI30 significantly facilitated spontaneous lung metastasis with more lung metastatic nodules, whereas knockdown of circIFI30 obviously inhibited pulmonary metastasis with fewer invasive tumor cells compared with control group (Figure 5E). Next, to assess the impact of circIFI30 on expression of target gene CD44 and epithelial-mesenchymal transition (EMT). IHC staining of CD44 and EMT-related markers (Twist, ZEB1 and E-cad) in tumor tissues was implemented. The results showed that upregulation of circIFI30 could enhance the expressions of CD44, Twist and ZEB1 as well as decrease the expression of E-cad in xenograft tumor tissues, whereas knockdown of circIFI30 played an opposite role compared with the control group (Figure 5F). Together, these data verified the oncogenic role of circIFI30 in TNBC.

### circIFI30 functions as an efficient miR-520b-3p sponge in TNBC

**Figure 6:**
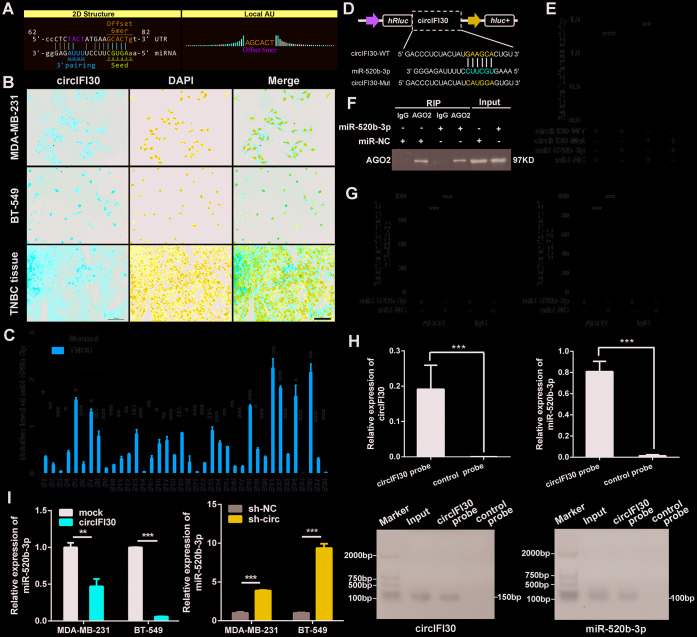
**circIFI30 functions as a sponge for miR-520b-3p.** (**A**) The miR-520b-3p binding site on circIFI30 was predicted by targetScan and miRanda. (**B**) FISH was performed to observe the cellular location of circIFI30 in TNBC cells (magnification, × 200, scale bar, 50 μm) and tissues (magnification, × 100, scale bar, 50 μm). (**C**) Relative expression of miR-520b-3p in TNBC tissues and adjacent non-tumor tissues was determined by qRT-PCR (n = 38). (**D**) Schematic illustration of circIFI30-WT and circIFI30-Mut luciferase reporter vectors was shown. (**E**) The relative luciferase activities were detected in 293 T cells after transfection with circIFI30-WT or circIFI30-Mut and miR-520b-3p mimics or miR-NC, respectively. (**F**, **G**) Anti-AGO2 RIP was executed in MDA-MB-231 cells after transfection with miR-520b-3p mimic or miR-NC, followed by western blot and qRT-PCR to detect AGO2 protein, circIFI30 and miR-520b-3p, respectively. (**H**) RNA pull-down with a biotin-labeled circIFI30 probe was executed in MDA-MB-231 cells, followed by qRT-PCR and RT-PCR to detect the enrichment of circIFI30 and miR-520b-3p. (**I**) The relative expression of miR-520b-3p was detected by qRT-PCR after transfection with indicated vectors. Data were indicated as mean ± SD, *P < 0.05, **P < 0.01, ***P < 0.001.

To explore the molecular mechanism underlying circIFI30, we first predicted the potential targets of circIFI30 using Arraystar’s miRNA target prediction software based on TargetScan and miRanda. The results indicated that circIFI30 had a putative conserved target site for miR-520b-3p (Figure 6A). Next, the subcellular localization of circIFI30 was detected in TNBC cells and tissues with FISH assay. We found that circIFI30 was mainly located in the cytoplasm of TNBC cells (Figure 6B). Then, the expression of miR-520b-3p was determined in 38 pairs of TNBC tissues and matched adjacent normal tissues with qRT-PCR, the data showed that miR-520b-3p was significantly downregulated in TNBC tissues (Figure 6C). Subsequently, dual-luciferase reporter assays were executed to measure the binding of circIFI30 with miR-520b-3p. Luciferase reporters with the wild type circIFI30 sequence (WT) or the sequence with mutated binding sites of miR-520b-3p (Mut) were constructed (Figure 6D). The data revealed that miR-520b-3p mimics significantly decreased the luciferase activity of circIFI30-WT luciferase reporter but not that of the mutant one (Figure 6E). It is well known that miRNAs inhibit the expression of target genes by binding to their 3′ UTR in an Argonaute 2 (AGO2)-dependent manner. Therefore, an anti-AGO2 RIP was carried out to pull down the the RNA transcripts combined with AGO2 in MDA-MB-231 cells, and IgG was used as negative control. As expected, circIFI30 and miR-520b-3p were effectively pulled down using anti-AGO2 antibody and were highly enriched in cells transfected with miR-520b-3p mimics compared with the controls (Figure 6F, 6G). In order to further confirm the binding of circIFI30 with miR-520b-3p, we used the specific biotin-labeled circIFI30 probes to conduct RNA pull-down analysis in circIFI30 overexpressing MDA-MB-231 cells. Results showed a specific enrichment of circIFI30 or miR-520b-3p in the circIFI30 probe group by qRT-PCR and PCR compared with the control group (Figure 6H). In addition, we found that upregulation of circIFI30 resulted in a significantly decrease of miR-520b-3p and downregulation of circIFI30 obviously enhanced the expression of miR-520b-3p in TNBC cells (Figure 6I). Altogether, our results demonstrated that circIFI30 could function as a sponge for miR-520b-3p in TNBC.

### MiR-520b-3p reverses the oncogenic effect of circIFI30 on TNBC cells in vitro

**Figure 7:**
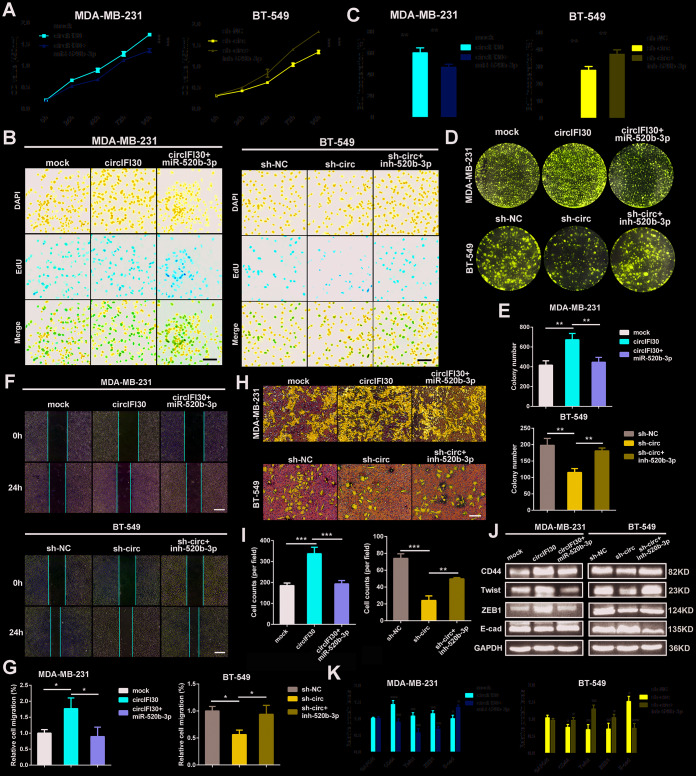
**circIFI30 promotes cell proliferation, migration and invasion through circIFI30/miR-520b-3p/CD44 axis.** (**A**) The cell viability was determined after transfection with indicated vectors, miR-520b-3p mimics or inhibitors by CCK8 assay. (**B**, **C**) The cell proliferation was detected after transfection with indicated vectors, miR-520b-3p mimics or inhibitors by EdU assay (magnification, × 100, scale bar, 100 μm). (**D**, **E**) The cell survival was measured after transfection with indicated vectors, miR-520b-3p mimics or inhibitors by colony formation assay. (**F**, **G**) The cell migration capacity was detected after transfection with indicated vectors, miR-520b-3p mimics or inhibitors by wound healing assays (magnification, × 50). Scale bar, 200 μm. (**H**, **I**) The cell invasion ability was determined after transfection with indicated vectors, miR-520b-3p mimics or inhibitors by transwell assays (magnification, × 100, scale bar, 100 μm). (**J**, **K**) Relative expressions of CD44 and EMT-related molecules at protein level in cells transfected with indicated vectors, miR-520b-3p mimics or inhibitors were determined by western blot. Data were indicated as mean ± SD, *P < 0.05, **P < 0.01, ***P < 0.001.

To probe whether circIFI30 plays its biological role via circIFI30/miR-520b-3p/CD44 axis, a series of rescue experiments were implemented. The results revealed that ectopic expression of miR-520b-3p markedly attenuated the proliferation, migration and invasion-enhancing roles mediated by upregulation of circIFI30 in TNBC cells, while miR-520b-3p inhibitors could counteract the inhibitory impacts of circIFI30 downregulation on TNBC cells proliferation, migration and invasion by CCK-8, EdU, colony formation, wound healing and transwell assays (Figure 7A–7I). Moreover, western blot analysis showed that overexpression of circIFI30 increased the expressions of CD44, Twist and ZEB1 as well as decreased the level of E-cad, whereas downregulation of circIFI30 played contrary roles in TNBC cells. MiR-520b-3p mimics or inhibitors could reverse the effects caused by overexpressing or silencing circIFI30, respectively (Figure 7J, 7K). Collectively, these results demonstrated that circIFI30 might function as a ceRNA for miR-520b-3p, which could contribute to EMT and progression of TNBC.

### CD44 is a direct target of miR-520b-3p and circIFI30 promotes TNBC development through circIFI30/miR-520b-3p/CD44 axis

**Figure 8:**
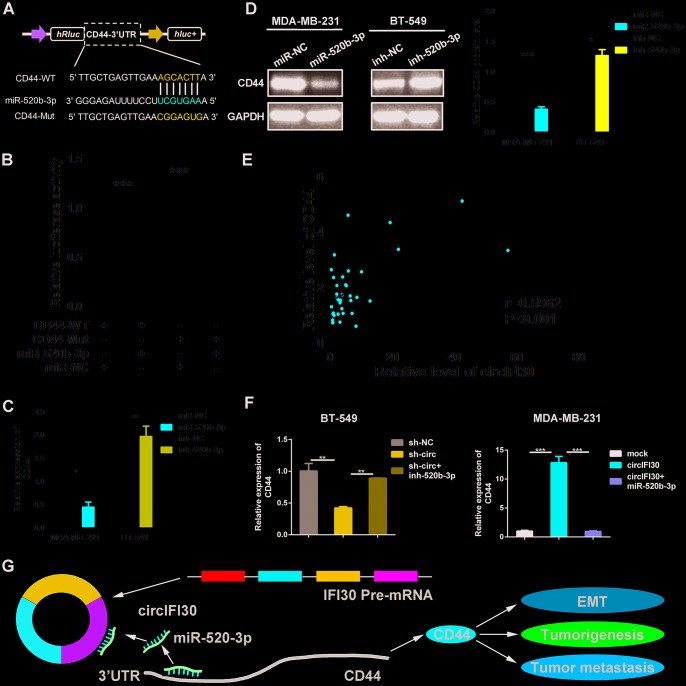
**CD44 is directly targeted by miR-520b-3p and regulated by circIFI30.** (**A**) Schematic illustration of CD44 3’UTR-WT and CD44 3’UTR-Mut luciferase reporter vectors was shown. (**B**) Luciferase reporter assay demonstrated that CD44 is direct target of miR-520b-3p. (**C**, **D**) Relative mRNA and protein levels of CD44 were detected after TNBC cells were transfected with miR-520b-3p mimics or inhibitors using qRT-PCR and western blot, respectively. (**E**) Pearson correlation analysis between the expression of circIFI30 and CD44 was shown in 38 TNBC tissues. (**F**) Relative expression of CD44 was detected by qRT-PCR in cells transfected with indicated vectors, miR-520b-3p or inhibitors. (**G**) The schematic diagram illustrates how circifi30 might promote EMT, tumorigenesis and metastasis of TNBC through circIFI30/miR-520b-3p/CD44 axis. Data were indicated as mean ± SD, *P < 0.05, **P < 0.01, ***P < 0.001.

To search the possible target of miR-520b-3p, bioinformatics analysis was executed utilizing the Targetscan (http://www.targetscan.org), miRanda (http://www.microrna.org/microrna/getDownloads.do) as well as FindTar software. The data showed that CD44 contains conserved target site of miR-520b-3p. The results of dual luciferase reporter assay showed that the activity of luciferase reporter vector with CD44 3’UTR-WT was significantly reduced by miR-520b-3p mimics compared to control groups (Figure 8A, 8B). Next, the expression of CD44 was determined in the 38 pairs of TNBC and adjacent non-cancerous tissues by qRT-PCR. We found CD44 was significantly up-regulated in TNBC tissues (Supplementary Figure 2). Moreover, miR-520b-3p mimics could markedly enhance the expression of miR-520b-3p, while miR-520b-3p inhibitors significantly reduced the level of miR-520b-3p in TNBC cells (Supplementary Figure 3). The qRT-PCR and western blot analysis showed that the expression of CD44 was notably reduced at both mRNA and protein levels in TNBC cells transfected with miR-520b-3p mimics, whereas CD44 expression was remarkably enhanced in TNBC cells transfected with miR-520b-3p inhibitors (Figure 8C, 8D). Furthermore, Pearson correlation analysis revealed that the expression of CD44 was positively related with the level of circIFI30 (Figure 8E). The qRT-PCR indicated that upregulation or downregulation of circIFI30 markedly promoted or suppressed the expression of CD44, and the effects could be reversed by miR-520b-3p mimics or inhibitors, respectively (Figure 8F). These data suggested that miR-520b-3p might directly target CD44 and circIFI30 could serve as a ceRNA for miR-520b-3p to upregulate the expression of CD44, which promote EMT, tumorigenesis and metastasis of TNBC (Figure 8G).

## DISCUSSION

CircRNAs, a kind of endogenous noncoding RNAs, have attracted great interest in recent years. Numerous circRNAs have been discovered and characterized with advances of next-generation sequencing technology and bioinformatics. Emerging evidences show that circRNAs play important roles in the tumorigenesis and cancer progression by acting as miRNA sponges, protein sponges, transcriptional regulators and protein-coding genes [17]. However, at present, only a few circRNAs have been well elucidated and the biological roles of most of them are still unclear. Searching for novel markers in patients with TNBC can predict the risk of the occurrence and metastasis and provide more effective targets for the diagnosis and therapy of TNBC.

In this study, we utilized RNA-seq to investigate the expression profile of circRNA in 4 pairs of TNBC tissues and adjacent normal tissues. Next, we characterized a new circRNA named circIFI30 which was remarkably up-regulated in TNBC and significantly related with TNM stage and overall survival of TNBC patients. Subsequently, *in vitro* and *in vivo* experiments showed that circIFI30 facilitated TNBC cell proliferation, motility, invasion and metastasis, whereas circIFI30 knockdown exhibited the contrary effects. We demonstrate that circIFI30 might act as a ceRNA to upregulate CD44 expression through sponging miR-520b-3p, which promote EMT, tumorigenesis and metastasis of TNBC. Our data suggest that circIFI30 might play an oncogenic role in pathogenesis and progression of TNBC.

The ceRNA hypothesis proposes that RNA transcripts, both coding and non-coding, can regulate each other by competing for the same miRNA response elements (MREs), which build a novel complex regulatory network and mechanism of interaction among RNA transcripts at the post-transcription level [10]. Increasing evidence showed that some circRNAs could act as sponges of miRNAs to regulate the expression of target genes in various cancers. For example, hsa_circ_0003998 serves as a ceRNA for miR-326 to facilitate cell proliferation and invasion of non-small cell lung cancer [18]. Circular RNA MYLK regulates VEGFA/VEGFR2 signaling pathway and promotes cancer progression through serving as a ceRNA of miR-29a in bladder cancer [19]. Besides, circPRKCI acts as a ceRNA to promote proliferation and tumorigenesis via sponging miR-545 and miR-589 and relieve the inhibition on the target gene E2F7 in lung adenocarcinoma [20]. In addition, circTADA2As inhibits development and metastasis of breast cancer through targeting miR-203a-3p/SOCS3 axis [21]. The circKIF4A-miR-375-KIF4A axis modulates TNBC development through ceRNA mechanism [22]. In the present study, bioinformatics analysis revealed that circIFI30 contained binding site of miR-520b-3p. We found that circIFI30 was located in cytoplasm of TNBC cells by nuclear-cytoplasmic fractionation and FISH assays. Dual-luciferase reporter, RNA pull-down and RNA immunoprecipitation assays further confirmed that circIFI30 could directly bind to miR-520b-3p. Thus, we supposed that circIFI30 could exert an oncogenic role through sponging miR-520b-3p in TNBC progression.

Recent research suggested that circRNAs could be important posttranscriptional regulators [23]. CircRNA could function as a ceRNA to regulate the expression of target genes of miRNA in light of the ceRNA hypothesis. Bioinformatics analysis indicated that CD44 is a possible target of miR-520b-3p using miRanda, FindTar and TargetScan. Next, we demonstrated that CD44 was remarkably upregulated in the TNBC tissues. Moreover, our data verified that miR-520b-3p might directly target the 3′-UTR of CD44 by dual-luciferase reporter assay. In addition, ectopically expressing miR-520b-3p could cause downregulation of CD44 at both mRNA and protein levels, whereas miR-520b-3p inhibitor revealed an opposite role, indicating that CD44 was a direct target of miR-520b-3p in TNBC. We further found that miR-520b-3p was significantly down-regulated in TNBC tissues. It has been shown that miR-520b-3p can act as a tumor suppressor by inhibiting cell proliferation and migration and is frequently downregulated in many types of cancer. MiR-520b-3p could target epidermal growth factor receptor (EGFR), histone deacetylase 4 (HDAC4) and calpain small subunit 1 (CAPN4) respectively to exert tumor-suppressive effects in gastric cancer, lung cancer, prostate cancer [24–26]. These reports were consistent with our finding. Furthermore, Pearson correlation analysis revealed that the level of CD44 was positively related with the expression of circIFI30. To further verify the interaction between circIFI30 and CD44, we demonstrated that up-regulation of circIFI30 could increase the expression of CD44 and promote proliferation, invasion and EMT in TNBC cells, whereas circIFI30 knockdown displayed contrary effects. The effects could be counteracted by miR-520b-3p mimic or inhibitor, respectively. Our results suggest that circIFI30 could function as a sponge for miR-520b-3p to relieve miRNA repression for target gene CD44 in TNBC.

Cell adhesion molecules (CAMs) are crucial in tumor development, and play a significant role in cell-cell communication as well as the adhesion between cells and extracellular matrix. CD44, a member of the CAM family, is a transmembrane glycoprotein that involves in cell proliferation, differentiation, adhesion and migration [27, 28]. CD44 is a widely distributed cell surface marker, which is used to identify and enrich tumor stem cells in different types of cancer including breast, colon, liver, ovarian, pancreatic cancers [29–31]. Especially, CD44 is considered as a dependable marker for breast cancer stem cells (BCSCs) and plays a significant role in invasion and metastasis of tumor. CD44 overexpression was correlated with a poor prognosis of TNBC patients [32]. Epithelial mesenchymal transition (EMT) contributes to tumor initiation, invasion, metastasis and drug resistance. The hyaluronic acid (HA, a proteoglycan, can be used as a scaffold for ECM assembly. HA-CD44 could interact and activate ZEB1, which is the key transcription factor promoting EMT [33]. Our previous research showed that up-regulation of circAGFG1 could enhance stemness of TNBC cells by increasing CD44 expression, whereas circAGFG1 knockdown played a reverse role [34]. These findings further support our results.

In conclusion, our results demonstrate that the expression of circIFI30 is up-regulated in TNBC and associated with the poor prognosis of TNBC patients. We also prove that circIFI30 might be a new oncogene in TNBC and reveal a novel ceRNA regulatory pathway in which circIFI30 upregulates CD44 expression through sponging miR-520b-3p to promote EMT, pathogenesis and metastasis of TNBC. Our data suggest that circIFI30 might be a promising prognosis marker and valuable therapy target for TNBC patients in the future.

## MATERIALS AND METHODS

### Ethical statement

The present investigation was conducted in accordance with the ethical standards and the Declaration of Helsinki and approved by the Ethics Committee of Chongqing Medical University. Informed consent from the patients has been obtained. Animal experiments were carried out under the approval of the Animal Ethics Committee of Chongqing Medical University. All the efforts were made to minimize the animal suffering.

### Tissue samples and cell culture

38 pairs of samples of TNBC tissues and adjacent normal tissues including 4 pairs of samples for RNA-seq were obtained from the TNBC patients treated in the First Affiliated Hospital of Chongqing Medical University (Chongqing, China) during 2016 to 2019. Tissue specimens were stored in liquid nitrogen until RNA extraction. MDA-MB-231, MDA-MB-468 and BT-549 human TNBC cell lines and MCF-10A normal mammary epithelial cell line were bought from American Type Culture Collection (ATCC) (Manassas, VA, USA). 293T cell lines were preserved in our laboratory. BT-549 cells were grown in RPMI 1640 medium (Gibco, Carlsbad, CA, USA) and other TNBC cell lines and 293T cells were maintained in DMEM medium (Gibco, Carlsbad, CA, USA) with 10% fetal bovine serum (Gibco, Carlsbad, CA, USA). MCF-10A cells were cultivated with MEBM BulletKit (Lonza, Basel, Switzerland). The cells were incubated in a humidified atmosphere containing 5% CO_2_ at 37°C.

### RNA sequencing

The total RNA was extracted using Trizol method (Takara, Dalian, China). Nanodrop 2000 spectrophotometer (Thermo Fisher Scientific, USA) and Agilent 2100 Bioanalyzer (Agilent Technologies, CA, USA) were applied to examine the quality, quantity and the integrity of RNA. The rRNA was removed from the purified RNA with RiboZero rRNA Removal Kit (Epicentre, WI, USA). Then, the RNA samples were randomly divided into small fragments and cDNA was synthesized with random primer. Purification of the PCR products of cDNA were conducted with AMPure XP Kit (Beckman Coulter, CA, USA). Next, quality control of the libraries and sequencing was executed by HiSeq2500 (Illumina, San Diego, USA).

### RNA extraction, qRT-PCR, nuclear-cytoplasmic fractionation, RNase R treatment and nucleic acid electrophoresis assays

Isolation of total RNA of tissues or cell lines was performed using TRIzol reagent (Takara, Dalian, China), and then reverse transcribed into cDNA, qRT-PCR analysis was executed by a Bio-Rad CFX96 system (Bio-Rad, CA, USA). GAPDH and U6 were used as internal controls respectively. The primers used are listed in Supplementary Table 1. The relative gene expression was calculated with 2 ^–ΔΔCT^ method. RNAs from nucleus and cytoplasm of TNBC cells were separated by the PARIS™ Kit (Life Technologies, Austin, Texas, USA) following the manufacturer's instructions. RNase R treatment was executed at 37 °C with 4 U/μg of RNase R (Epicentre Biotechnologies, Madison, WI, USA) for 30 min. The cDNA and Genomic DNA (gDNA) of circIFI30 and GAPDH from TNBC cells were amplified by divergent primers and convergent primers, respectively. PCR products were detected with 2% agarose gel electrophoresis at 90 V for 40 min. The bands were observed by UV irradiation.

### In situ hybridization (ISH) of tissue microarray (TMA)

In situ hybridization with a specific digoxin-labeled circIFI30 probe (Digoxin-5’- GGTATAGATTGCCTGTTAGTGGCAGACTTCTCT-3’-Digoxin, Geneseed, Guangzhou, China) was used to detect the relative expression of circIFI30 in 78 TNBC samples on TMAs (Outdo Biotech, Shanghai, China). Briefly, after dewaxed and rehydrated with gradient alcohol, the tissues were digested by proteinase K, fixed with 4% paraformaldehyde, then hybridized overnight by the digoxin-labeled circIFI30 probe at 4°C, then incubated overnight using an anti-digoxin-AP (Roche, Basel, Switzerland) at 4°C. The samples were stained by NBT/BCIP (Roche, Basel, Switzerland), observed and quantified. The ISH staining score was calculated by multiplying the value for intensity of positive staining (negative = 0, weak = 1, moderate = 2 and strong = 3) and the proportion of positively stained cells (<10%=0, 10-25%=1, 26-50%=2, 51-75%=3, >75%=4). The ISH score <6 indicated low expression, while ≥6 defined high expression.

### Vector construction and cell transfection

The full-length of human circIFI30 sequence was inserted into the pLCDH-ciR vector (Geenseed Biotech, Guangzhou, China) to construct overexpression vector, and siRNAs targeting back splice junction of circIFI30 (si-RNA1, si-RNA2, siRNA-3) were synthesized (Geenseed Biotech, Guangzhou, China) for knockdown of circIFI30. The efficiency of siRNA was evaluated by qRT-PCR (Supplementary Figure 1). SiRNA-3 was selected as the most effective one for synthesizing shRNA, and then the synthesized shRNA and negative control shRNA-NC were subcloned into the pLL3.7 vector to construct RNAi vector, termed as sh-circ and sh-NC, respectively. These vectors were confirmed with sequencing. Lentiviruses carrying sh-circ and sh-NC, named as lv-sh-circ and lv-sh-NC, were purchased from Hanbio Biotechnology (Shanghai, China) for animal experiments. The miR-520b-3p mimics and inhibitors were bought from GenePharma (Shanghai, China). The transfections were implemented using Lipofectamine 2000 (Invitrogen, Carlsbad, CA, USA) according to the manufacturer’s instructions. The sequences of siRNAs and shRNAs were indicated in Supplementary Table 2.

### Cell proliferation, cell cycle and apoptosis assays

The cell proliferation and viability were examined using CCK-8, EdU and colony formation assays. Cell Counting Kit-8 was purchased from Bosterbio (Wuhan, China), 2000 cells/well were inoculated in 96-well plates with complete medium, added 10 μl CCK-8 per well, then incubated at 37°C for 2 h. After incubation for 24, 48, 72 and 96 h, respectively, the absorbance value at 450 nm was recorded by a plate reader (Bio-Rad, Hercules, CA). For EdU assay, 1×10^5^ cells were inoculated to 24-well plates using EdU cell proliferation kit (Ribobio, Guangzhou, China). The percentage of EdU-positive cells was counted in four random fields per well. 2.5×10^3^ cells were added into 6-well plates for colony formation assay and cultured for two weeks, fixed by 4% paraformaldehyde, stained using 0.5% crystal violet. The images were captured and the number of clones was counted. For cell cycle analysis, 2×10^6^ cells were fixed with 70 % ethanol for 12 h. The cell cycle analysis was performed using flow cytometry (Becon Dickinson FACSCalibur, NY, USA). The cells were fixed with 4% paraformaldehyde and dyed by Hoechst 33342, then observed under fluorescence microscope (Leica, Wetzlar, Germany). Apoptosis was detected by TUNEL Apoptosis Assay Kit (Beyotime, Shanghai, China). The apoptotic cells labeled with FITC were viewed under fluorescent microscope. The percentage of early apoptotic cells was detected by flow cytometry (Becon Dickinson FACSCalibur, USA) with annexin V-FITC/PI double staining.

### Wound healing and invasion assays

TNBC cells were inoculated into 6-well plate and scratches were made with a 200 μl tip at 24 hours post transfection, and then were incubated with serum-free medium, the wound width was measured at three separate wound sites and normalized to the width of control group after 24 hours. The cell invasion experiments were implemented with matrigel-coated transwell chambers (BD BioCoat, Bedford, MA, USA). 2×10^4^ cells in 200 μl serum-free medium were placed in the upper chamber, and then added 500μl complete medium to the bottom chambers. After 24 h, the non-invading cells in the upper compartment were erased and invading cells in the lower chambers were stained using crystal violet. Finally, the cells were taken photos and quantified under a microscope (Leica, Wetzlar, Germany).

### Animal experiments

Female BALB/c mice (4-6 weeks old) were subcutaneously inoculated with 2×10^6^ stably transfected or infected MDA-MB-231 cells. Tumor volume was monitored once a week and estimated by 0.5×length×width^2^. After 4 weeks, the mice were killed and the lungs and tumors were excised for further research. Metastatic nodules of the lung were counted under microscope. The microvessels were counted on HE-stained slides from the tumors under microscope corresponding to areas with the highest vascular density.

### Immunohistochemistry (IHC)

For IHC staining, after dewaxing, rehydration, and antigen retrieval, the sections were incubated with primary antibodies against CD44, Twist, ZEB1 and E-cad (1:100) (Abcam, Burlingame, CA, USA) at 4 °C overnight, then incubated for 2 h at 37 °C with secondary antibodies. Subsequently, HRP-labeled streptavidin solution was added to the slices for 15 min, then slides were stained by DAB and counterstained with hematoxylin. Finally, observation was performed under a microscope (Leica, Wetzlar, Germany).

### Fluorescence in situ hybridization (FISH)

The subcellular localizations of circIFI30 and miR-520b-3p in TNBC cells were observed with FISH. Concisely, after prehybridization at 55°C for 2h, frozen tissue sections and cell slides were hybridized with specific Cy3-labeled circIFI30 probes (Cy3-5’-GGTATAGATTGCCTGTTAGTGGCAGACTTCTCT-3’-Cy3) (Geneseed, Guangzhou, China) at 37°C overnight. Slides were dyed with DAPI and photographed under a fluorescence microscope (Leica, Wetzlar, Germany).

### Dual-luciferase reporter assay

The sequences of circIFI30 and CD44-3’UTR and their mutants without miR-520b-3p binding sites were produced and inserted into luciferase reporter vector psiCHECK2 (Promega, Madison, WI, USA), named circIFI30-WT, circIFI30-Mut, CD44 3’UTR-WT and CD44 3’UTR-Mut, respectively. The plasmids were identified by sequencing. The relative luciferase activity was detected using Dual Luciferase Assay Kit (Promega, Madison, WI, USA) following the manufacturer's instructions.

### RNA immunoprecipitation (RIP)

RIP was executed by Magna RIP kit (Millipore, Billerica, MA, USA) according to the manufacturer's protocol. MDA-MB-231 cells were transfected with miR-520b-3p mimics or miR-NC. After 48h, the cells were lysed with RNA lysis buffer, then cell lysates were incubated with the RIP buffer containing magnetic beads conjugated to anti-Argonaute2 (AGO2) (Millipore, Billerica, MA, USA) or negative control IgG antibody (Millipore, Billerica, MA, USA) for 4 h at 4°C. After washing three times with washing buffer, western blot and qRT-PCR were implemented to detect enriched miR-520b-3p, circIFI30 and AGO2.

### RNA pull-down

The biotin-coupled circRNA pull-down assay was performed. Briefly, biotin-labeled circIFI30 probe (5’-GGTATAGATTGCCTGTTAGTGGCAGACTTCTCT-3’-Biotin) and control probe (5’-CCATATCTAACGGACATAGTGGCAGACTTCTCT-3’-Biotin) were produced by Geneseed Biotech. TNBC cells were lysed using lysis buffer and incubated by specific probes of circIFI30. To pull down the biotin-coupled RNA complex, the lysates from cells were incubated with streptavidin-coupled magnetic beads. The beads were washed three times with the buffer. The RNAs were extracted using TRIzol (Takara, Dalian, China). Then the abundance of circIFI30 and miR-520b-3p was determined with qRT-PCR and RT-PCR.

### Western blot analysis

Briefly, the proteins were extracted, quantified and isolated by 10% SDS-PAGE. Next, the separated protein bands were transferred to PVDF membranes (Bio-Rad, CA, USA). The membranes were blocked with 4% skim milk powder and incubated with primary antibody against CD44, Twist, ZEB1 and E-cad (1:1000) (Abcam, Burlingame, CA, USA), Bax, Bcl-2 and cleaved Caspase-3 (1:1000) and GAPDH (1:5000) (Cell Signaling Technology, Beverly, MA, USA) overnight at 4°C, followed by incubating with a secondary antibody (1:5000 dilution) (Cell Signaling Technology, Beverly, MA, USA) for 2 h and detected by chemiluminescence.

### Statistical analysis

Statistical analyses were executed by SPSS 21.0 (IBM, SPSS, Chicago, IL, USA) and GraphPad Prism 6.0 (GraphPad Software Inc., CA, USA). Data are presented as the mean±S.D (Standard Deviation). The differences between groups were analyzed using Student’s t test, one-way ANOVA or chi-square test. The survival analysis was assessed by Kaplan-Meier plots and log-rank tests. Multivariate Cox proportional hazards regression model were used to determine the effect of clinical variables on the overall survival rate of TNBC patients. Correlations were analyzed by Pearson’s correlation test.

## Supplementary Material

Supplementary Figures

Supplementary Tables
