# Supplementary material for: The circRNA circIFI30 promotes progression of triple-negative breast cancer and correlates with prognosis
Source: Aging (Albany NY). 2020 Jun 4;12(11):10983–1003. doi: 10.18632/aging.103311 (PMC7346060; doi:10.18632/aging.103311)
Supplement: Supplementary Figures [file aging-12-103311-s002..pdf]

## SUPPLEMENTARY FIGURES

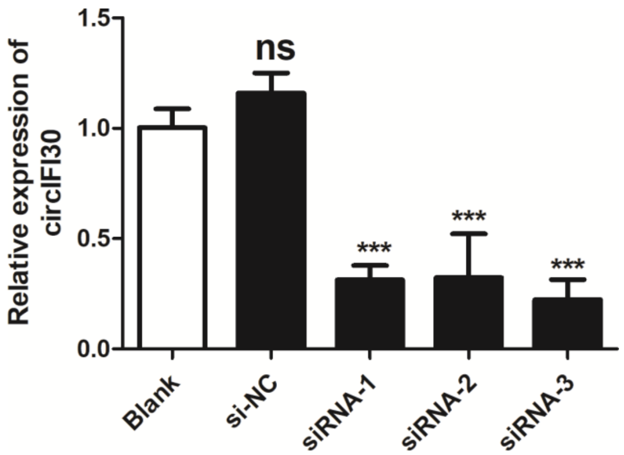


**Supplementary Figure 1. The efficiency of circIFI30 siRNA was evaluated by qRT-PCR.**

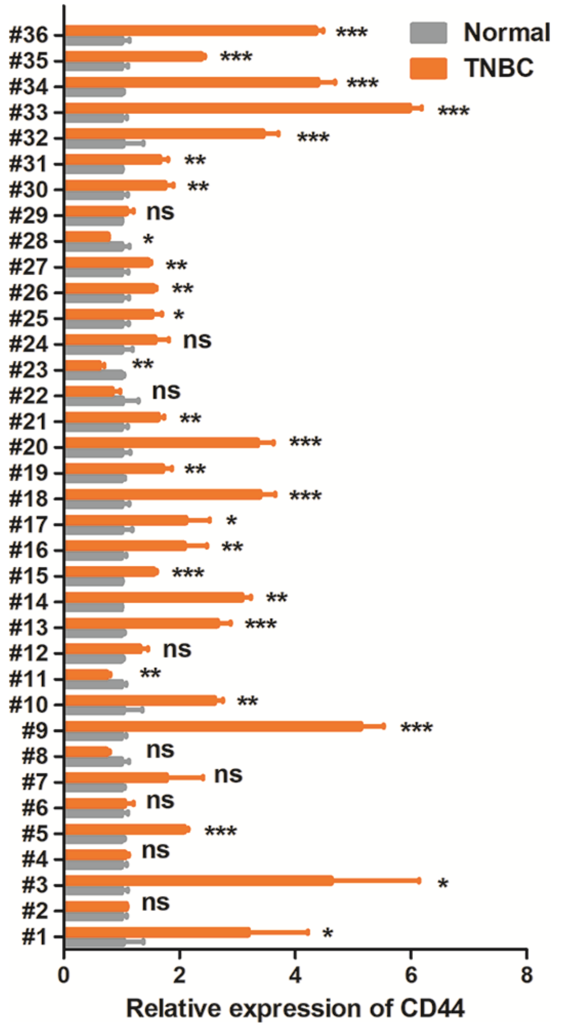


**Supplementary Figure 2. Relative expression of CD44 in TNBC tissues and adjacent non-tumor tissues was detected by qRT-PCR.**

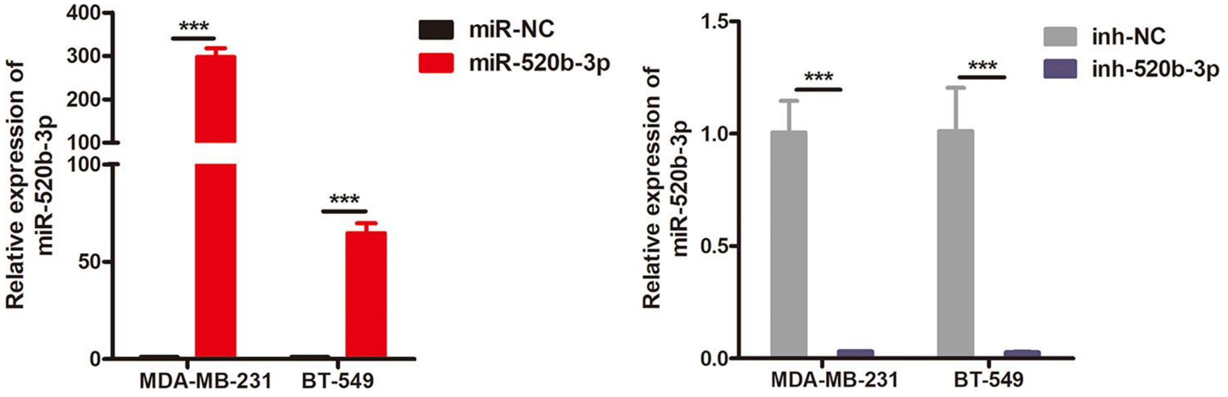


**Supplementary Figure 3. The miR-520b-3p expression level was determined in TNBC cells transfected with miR-520b-3p mimics and miR-520b-3p inhibitors by qRT-PCR.**
