# Supplementary material for: The circRNA circIFI30 promotes progression of triple-negative breast cancer and correlates with prognosis
Source: Aging (Albany NY). 2020 Jun 4;12(11):10983–1003. doi: 10.18632/aging.103311 (PMC7346060; doi:10.18632/aging.103311)
Supplement: Supplementary Tables [file aging-12-103311-s001..pdf]

SUPPLEMENTARY TABLES

Supplementary Table 1. Sequences of primers used in this study.

Gene	Primer sequences
circIFI30	F: 5’-GGAGTGCAAATTCAACAAGGT-3’
	R: 5’-AGGTATAGATTGCCTGTTAGTG-3’
CD44	F: 5’-CTGCCGCTTTGCAGGTGTA-3’
	R: 5’-CATTGTGGGCAAGGTGCTATT-3’
GAPDH	F: 5’-GAAGGTGAAGGTCGGAGTC-3’
	R: 5’-GAAGATGGTGATGGGATTTC-3’
miR-520b-3p	F: 5’- ATGGTTCGTGGGAAAGTGCTTCCTTTTAGAGG-3’
	R: 5’- GTGCAGGGTCCGAGGT-3’
	RT:5’- GTCGTATCCAGTGCAGGGTCCGAGGTATTCGCACTGGATACGACCCCCTCTAA-3’
U6	F: 5’-CTCGCTTCGGCAGCACA-3’
	R: 5’-AACGCTTCACGAATTTGCGT-3’
	RT: 5’-CTCGCTTCGGCAGCACAPCR-3’

Supplementary Table 2. Sequences of siRNAs and shRNAs used in this study.

Definition	sequences
siRNA-1	5’-AGAAGTCTGCCACTAACAG-3’
siRNA-2	5’-GCCACTAACAGGCAATCTA-3’
siRNA-3	5’-CACTAACAGGCAATCTATA-3’
si-NC	5’-TTCTCCGAACGTGTCACGT-3’
sh-circ	5’-GTTAACCACTAACAGGCAATCTATATCAAGAGTATAGATTGCCTGTTAGTGTTTTTTCTCGAG-3’
sh-NC	5’-TTCTCCGAACGTGTCACGTTCAAGAGACGTGACACGTTCGGAGAATTTTTT-3’
